# King’s Parkinson’s Disease Pain Questionnaire: reliability and convergent construct validity

**DOI:** 10.1590/0034-7167-2022-0379

**Published:** 2023-08-07

**Authors:** Ana Carolina Sartori, Fânia Cristina dos Santos, Juliana de Lima Lopes, Vinicius Batista Santos, Meiry Fernanda Pinto Okuno

**Affiliations:** I Universidade Federal de São Paulo. São Paulo, São Paulo, Brazil

**Keywords:** Parkinson’s Disease, Elderly, Pain, Validation Studies, Health Services., Enfermedad de Parkinson, Adulto Mayor, Dolor, Estudios de Validación, Servicios de Salud., Doença de Parkinson, Idoso, Dor, Estudos de Validação, Serviços de Saúde.

## Abstract

**Objectives:**

to assess the evidence of reliability and convergent construct validity of the King’s Parkinson’s Disease Pain Questionnaire.

**Methods:**

psychometric study of 75 older adults with Parkinson’s disease. The instrument was applied by two researchers separately and reapplied by one researcher 15 days later. In terms of reliability, internal consistency was assessed using the Cronbach’s alpha test and stability using the intraclass correlation coefficient. Scores of the King’s Parkinson’s Disease Pain Questionnaire were compared to those of the Geriatric Pain Measure in the assessment of construct validity.

**Results:**

the mean Cronbach’s alpha obtained between the three assessments was above 0.60, the intraclass correlation between the three assessments was above 0.90, and there was a weak but significant correlation between the two applied scales.

**Conclusions:**

the instrument showed adequate evidence of convergent construct validity and reliability, and can be used in clinical practice.

## RESUMO

**Objetivos:**

avaliar as evidências de confiabilidade e de validade de construto convergente do *King’s Parkinson’s Disease Pain Questionnaire*.

**Métodos:**

estudo psicométrico com 75 idosos com doença Parkinson. O instrumento foi aplicado por dois pesquisadores separadamente e reaplicado por um dos pesquisadores após 15 dias. Na confiabilidade, a consistência interna foi avaliada pelo teste de alfa de Cronbach e a estabilidade pelo coeficiente de correlação intraclasse. Na avaliação da validade de construto, os escores do *King’s Parkinson’s Disease Pain Questionnaire* foram comparados ao escore da *Geriatric Pain Measure.*

**Resultados:**

foi obtido um alfa de Cronbach médio entre as três avaliações acima de 0,60 e correlação intraclasse entre as três avaliações acima de 0,90, bem como uma correlação fraca, mas significativa entre as duas escalas aplicadas.

**Conclusões:**

o instrumento apresentou adequadas evidências de validade de construto convergente e de confiabilidade, podendo ser utilizado na prática clínica.

## RESUMEN

**Objetivos:**

evaluar las evidencias de confiabilidad y validez convergente de constructo del *King’s Parkinson’s Disease Pain Questionnaire*.

**Métodos:**

estudio psicométrico con 75 adultos mayores con enfermedad de Parkinson. El instrumento fue aplicado por dos investigadores por separado y vuelto a aplicar por uno investigador después de 15 días. En cuanto a la confiabilidad, se evaluó la consistencia interna mediante la prueba alfa de Cronbach y la estabilidad mediante el coeficiente de correlación intraclase. Al evaluar la validez del constructo, las puntuaciones del *King’s Parkinson’s Disease Pain Questionnaire* se compararon con las de la *Geriatric Pain Measure.*

**Resultados:**

se obtuvo un alfa de Cronbach promedio entre las tres evaluaciones por encima de 0,60 y correlación intraclase por encima de 0,90, y una correlación débil pero significativa entre las dos escalas aplicadas.

**Conclusiones:**

el instrumento mostró evidencia adecuada de validez convergente de constructo y confiabilidad, y puede ser utilizado en la práctica clínica.

## INTRODUCTION

The increasing life expectancy of the population leads to the emergence of several pathologies associated with a decrease in functional capacity, including Parkinson’s disease (PD). This chronic, progressive, idiopathic disease of the central nervous system (CNS) generally affects individuals over 65 years of age^(1)^. By 2040, it is expected that around 17 million people will be affected by PD, and according to the study of the global burden of diseases, compared to all other neurological diseases, PD is the one that grows the most in the world^(2)^.

Parkinson’s disease is characterized by a gradual loss of dopaminergic neurons in the substantia nigra. The dopamine neurotransmitter is a chemical that helps message transmission between nerve cells. The loss of these neurons causes motor and non-motor symptoms and affects the quality of life (QoL) of older adults, leading to social isolation and sadness. Motor symptoms include tremor at rest, muscle rigidity, sialorrhea and bradykinesia, while non-motor symptoms include anxiety, fatigue or depressed mood^(3)^.

Pain in patients with PD is another very important and little-reported symptom that can affect 40 to 85% of this population. It is usually associated with motor symptoms such as postural changes, and this manifestation may be a consequence of the disease and occur at the beginning, before the onset of motor difficulties^(4)^.

In PD, pain can be musculoskeletal and radicular/neuropathic, related to involuntary muscle contractions, pain/discomfort caused by restlessness and central pain. This pain begins to direct and limit patients’ decisions and behavior, as it causes physical and functional disability, directly affecting QoL^(5)^. Adequate assessment of pain in this population is one of nurses’ role, so that interventions can be directed to achieve positive health outcomes^(6)^.

Pain, which is often disabling, must be adequately assessed and quantified, mainly through instruments with adequate evidence of validity. In Brazil, the only scale aimed at the assessment of neuropathic pain among older adults with PD is the King’s Parkinson’s Disease Pain Questionnaire (KPPS), through which is possible to characterize the pain (degree and location) and identify the patient’s needs for the planning of care by nursing and the multidisciplinary team. Once put into practice, it can minimize the pain and consequently the loss of functional capacity, and prevent complications in older adults with PD^(7)^.

The KPPS questionnaire was developed by researchers in the UK, Germany, France, Romania, Sweden, Italy and Spain, with funding from various institutes and foundations linked to departments of health and Parkinson’s. Even though this instrument has been translated and adapted into Brazilian Portuguese in a previous study^(8)^, other analyzes of evidence of validity in the Brazilian population have not been performed.

## OBJECTIVES

To assess the evidence of reliability and convergent construct validity of the King’s Parkinson’s Disease Pain Questionnaire.

## METHODS

### Ethical aspects

The present study was evaluated and approved by the Research Ethics Committee of the *Universidade Federal de São Paulo*, and a declaration of consent for validation was signed by the author of the instrument.

### Type of study

This is a psychometric study of reliability analysis and convergent construct validity.

### Study scenario

Data were collected from older adults with PD from the Neurology service, Movement Disorders Outpatient Clinic; and the Service of Pain and Osteoarticular Diseases, Discipline of Geriatrics and Gerontology - DIGG, both at the *Universidade Federal de São Paulo*, city of São Paulo.

### Data source

The study had a non-probabilistic convenience sample of older adults, totaling 75 participants. They met the following inclusion criteria: age 60 years or older, both sexes, with PD, diagnosed according to the Diagnostic and Statistical Manual (DSM-V), with chronic pain (six months or more), intensity greater than 3 on the visual analogue scale and any etiology. Patients with severe dementia and communication impairment were excluded.

### Data collection and organization

Sociodemographic and clinical characterization instruments containing the following variables were applied: age, sex, ethnicity/race, education, marital status, occupation, family income, number of dependents on the family income, time of diagnosis of Parkinson’s disease and the presence of comorbidities.

The KPPS was applied after the initial data collection. It comprises seven domains, namely, musculoskeletal pain (one question), chronic pain (two questions), intermittent pain (three questions), night pain (two questions), orofacial pain (three questions), discoloration/edema/swelling (two questions) and radicular pain (one question). Each item must be evaluated for severity on a 0-3 points scale; 0 is none, 1 mild, 2 moderate and 3 severe, multiplied by the frequency, which varies from 0 to 4 points; 0 is never, 1 rarely, 2 sometimes, 3 often and 4 very often. The score in each domain is used to determine the type of pain experienced by the patient, while the total score provides insight into the impact of pain on the individual’s life. The end of the scale results in the assessment of pain location, intensity and frequency, and the relationship between musculoskeletal pain and motor instability^(7)^. The partial result ranges from 0 to 12 for each domain item and the total score ranges from 0 to 168 points^(7-8)^.

The third instrument applied to assess the convergent construct validity was the Geriatric Pain Measure (GPM), which evaluates painful conditions. It is a quick-execution and simple-to-understand questionnaire with multidimensional qualities. It is used to assess older adults with chronic pain and the consequences of these pains on their mood, life activities and quality of life. Composed of 24 items, it has a total score obtained by the sum of item scores, ranging from 0 (zero pain) to 42 points (severe pain), and can be adjusted for a total score ranging from 0 to 100 (adjusted total score) by multiplying the sum of final scores by 2.38. The adjusted total score allows classifying pain as mild in a score of 0-30 points, moderate 30-69 points, and severe if the score is greater than 70^(8)^.

The GPM scale was developed by the Greater Los Angeles VA Geriatric Research Education and Clinical Center, the Jewish Home for the Aging, the VA/UCLA Multicampus Program of Geriatrics and Gerontology and the Swiss National Science Foundation^(9-10)^. The cross-cultural adaptation to Brazilian Portuguese was performed in a previous study^(8,11-12)^.

### Work steps

Data collection took place between January 2021 and January 2022. Older adults were initially invited to participate in the study and if they agreed, the informed consent form was given for signature. Sociodemographic and clinical characterization data were collected from patients’ medical records.

The KPPS instrument was applied by two researchers separately on the same day. An interviewer was one of the researchers in this study, and the second was a health professional from the Neurology Outpatient Clinic who received training on the study and how to apply the instrument. After applying the KPPS, one of the researchers applied the GPM. In a second moment, after an interval of at least seven days and a maximum of 15 days, the KPPS was reapplied by one of the researchers, making sure that no new analgesic intervention was performed during this period^(8)^.

Although the KPPS is a self-administered instrument, we chose to collect data in the form of an interview, considering participants’ difficulty in filling it out because of motor symptoms and the possibility of visual difficulties and/or low instructional level. When some degree of difficulty was found in understanding the meaning of a question, it was re-read slowly, avoiding giving synonyms or explanations to the words, and the same was done with the scale of answers. The average time to apply the questionnaires was 40 minutes.

### Data analysis

The Minitab 16, Excel Office 2010 and the SPSS version 20.0 were used in data analysis. Median and quartile values were calculated for quantitative variables as, according to the Komolgorov Sminorv test, all these variables violated the data normality assumptions. Qualitative data were expressed in absolute and relative frequency.

In the analysis of instrument reliability, internal consistency was calculated using the Cronbach’s alpha test; values above 0.7 were considered ideal, and 0.6-0.7 satisfactory. The intraclass correlation coefficient (ICC) was calculated for analysis between observers in the first collection and in relation to instrument stability. Values less than 0.5 were considered poor, 0.5-0.75 moderate, 0.75-0.90 good, and greater than 0.9 excellent^(13-14)^.

Another analysis performed for the three applications was the calculation of the SEM (standard error of measurement). It was used to calculate the MCID (Minimum Clinically Important Difference), which is the multiplication of the SEM by the square root of 2, multiplied by 1.96 (statistical probability with 95% confidence)^(13)^.

In the assessment of convergent construct validation, mean KPPS scores were compared to mean GPM values to obtain convergent validity data. This analysis was performed using the Spearman’s correlation test; correlation coefficients from 0.10 to 0.40 were considered as weak, 0.40 to 0.60 as moderate and above 0.60 as strong^(13)^.

A significance value of 5% was adopted in this study.

## RESULTS

The study included 75 older adults; most were female (51.5%), mean age of 69.6 years, white (48.3%), predominance of illiterates (53.8%), income of less than a minimum wage (51. 5%), with a partner (46.9%), retired (47.2%) and most had no other comorbidities (53.2%).

The specific region of the body with the most frequent pain was the lumbar spine (60%), followed by the leg (48%), knee (42.7%), arm (30.7%), shoulder (24%), foot (21.3%), thigh (18%), hand (12%). The most frequent type of pain was musculoskeletal (90.7%), followed by neuropathic radicular pain (26.7%), dystonia pain (24%), central pain (1.3%) and akathisia (1.3%).

In the KPPS analysis, a median value of 45 points was obtained in the three applications, and the lowest pain scores were from domains related to chronic pain, orofacial pain and radicular pain, as shown in Table 1.

**Table 1:** Median of the King’s Parkinson’s Disease Pain Questionnaire domains in the three assessments, São Paulo, São Paulo, Brazil, 2021

Domains	1^st^ Assessment Median (Q1;Q3)	2^nd^ Assessment Median (Q1;Q3)	3^rd^ Assessment Median (Q1;Q3)
1: Musculoskeletal pain (0-12 points)	9 (3;12)	8 (3;12)	9 (4;12)
2: Chronic pain (0-24 points)	0 (0;9)	0 (0;6)	0 (0;8)
3: Intermittent pain (0-36 points)	12 (2;24)	12 (3;24)	12 (2;21)
4: Night pain (0-24 points)	12 (3;12)	12 (0;15)	12 (0;12)
5: Orofacial pain (0-36 points)	0 (0;9)	0 (0;9)	0 (0;9)
6: Discoloration; edema/ swelling (0-4 points)	3 (0;9)	2 (0;9)	2 (0;9)
7: Radicular pain (0-12 points)	0 (0;8)	0 (0;6)	0 (0;6)

*Med - mediana; Q1 - primeiro quartil; Q3 - terceiro quartil.*

In the analysis of internal consistency of the instruments, a Cronbach’s alpha value of 0.69 was obtained in the first application, 0.72 in the second, 0.67 in the third, and an ICC of 0.99 (p<0.01) in the analysis between the two interviewers in the first application of the instrument. After 15 days, on average, the instrument was reapplied to assess its stability, obtaining an ICC of 0.99 when compared to the application between the first and second interviewers.

Comparing the median values obtained in the three assessments, there was no statistically significant difference between measures, as shown in Table 2. By evaluating the minimum clinically important value in the three assessments, an average value of 9 points was obtained in the score to be considered by the professional, according to Table 2.

**Table 2:** Comparison between the three applications of the King’s Parkinson’s Disease Pain, São Paulo, São Paulo, Brazil, 2021

KPPS	1^st^ Application	2^nd^ Application	3^rd^ Application	*p* value^*^
Median	45	45	45	0.60
Q1	24	24	22.5	
Q3	70	71	68.5	
CI	6.53	6.68	6.58	
SEM	3.33	3.41	3.36	NA
MCID	9.23	9.45	9.3	NA

CI - confidence interval; SEM - standard measurement error; MCID - Minimum Clinically Important Difference;

* Friedman’s test; NA - does not apply.

In the analysis of the convergent construct validity based on the comparison between the mean KPPS score and the mean GPM score (comparing the three measures), a weak but significant correlation coefficient was obtained, as shown in Table 3.

**Table 3:** Spearman’s Correlation Coefficient between the Geriatric Pain Measure and the King’s Parkinson’s Disease Pain, São Paulo, São Paulo, Brazil, 2021

		KPPS 1^st^ Application	KPPS 2^nd^ Application	KPPS 3^rd^ Application
GPM	R	0.323	0.331	0.318
	*p* value	0.005	0.004	0.005

*R - Spearman correlation coefficient.*

## DISCUSSION

Pain is a frequently ignored symptom mainly due to its still poorly understood processes, which contributes to the lack of adequate symptomatic management in PD therapy. The need for research that allows the clarification and adequate assessment of this symptom is evident, especially in preliminary stages of the disease, as a better understanding may have a positive impact on the identification of signs for an early diagnosis and a more satisfactory treatment of PD^(15)^.

Regarding the prevalence of PD, although the literature does not indicate a difference in its incidence between sexes, most individuals in the study were female. A greater tendency for occurrence has been identified among men, probably due to the attribution of cultural aspects, given the physical and emotional stress that they suffer during life. There are studies showing the consequences of estrogen neuroprotectors throughout life, which would be a possible reason for such data, but the role of estrogen as a neuroprotector is still controversial. In India, for example, where a majority of men was observed among people with PD, the authors themselves conceded the fact to the cultural and social context, which prevents women from seeking health services^(16)^.

In this study, most respondents had 1-4 years of schooling. People with low education tend to be slower readers, have difficulties in interpreting audiovisual messages, a worse performance in language and consequently, perform more poorly in cognitive tasks. All the aforementioned factors make these older adults less resistant to the progression of a neural, progressive disease such as Parkinson’s, since there is no constant stimulation of the brain areas that would help them to cope with it^(17-18)^.

Pain in PD can be of musculoskeletal origin; radicular/neuropathic; related to dystonia; pain/discomfort as a result of akathisia (restlessness) and central pain. The most cited etiology by patients in the present study was of musculoskeletal origin and is associated with stiffness caused by the disease itself or akinesias^(19)^.

In another study, the region of pain more cited by the patients studied was the lumbar region, being considered one of the most affected body segments in subjects with PD and with multifactorial repercussions. In addition to pain itself, there are more extensive effects, such as activity limitations, participation restrictions, caregiver burden, use of medical care resources and financial burden^(20)^.

Although most of those surveyed did not have other comorbidities, the literature shows that the presence of depression and other clinical diseases is very constant. As a result, there is a worse evolution of both the psychiatric condition and the clinical disease, with less acceptance of therapeutic guidelines and higher morbidity and mortality^(21)^.

In this study, a median value of 45 points in the three applications was obtained. The domains with lower pain scores were related to chronic pain, orofacial pain and radicular pain, similar to another KPPS scale validation study of the Bulgarian population, in which a relationship was also identified between domains of lower pain scores and chronic pain; discoloration, edema/swelling and orofacial pain. The most common type of pain is musculoskeletal, regardless of the instrument used to assess specific pain for PD^(22)^.

The achieved values of internal consistency ranged from 0.67 to 0.72 and an ICC of 0.99, similar to the values achieved in the original study (Cronbach’s alpha 0.70)^(7)^. In a study that examined the psychometric properties of the Persian version of the KPPS, Cronbach’s alpha and ICC values were greater than 0.80, findings that partially corroborate what was found in this study, in which values of 0.67-072 were obtained in the analysis of the internal consistency of the instrument and an ICC of 0.99^(23)^.

In a study in Bulgaria, the Cronbach’s alpha value obtained in 162 patients with idiopathic Parkinson’s disease was 0.75 and in the stability analysis using the ICC, the value was 0.92, very similar to data found in the current study (ICC 0.99)^(22)^.

In another multicenter study with the objective of analyzing the evidence of convergent construct validity between the King’s Parkinson’s Disease Pain Questionnaire (KPPQ) and the KPPS, a strong correlation (r=0.80) was identified between the two instruments. In this study, in the analysis of evidence of convergent construct validity, the objective was to compare two instruments that assessed the pain construct, and a weak (r=0.331) but significant correlation coefficient was obtained in the three measures^(24)^. It could be reapplied to a larger population sample in order to assess if there is an increase in this correlation.

The GPM was developed to allow a multidimensional assessment of pain. It addresses multiple dimensions of pain, such as intensity, pain when walking, pain in vigorous activities and pain in other activities, including sensory-discriminative, motivational-affective and cognitive dimensions. As the KPPS instrument evaluates the specific pain caused by PD, this may be the reason for identifying a weak correlation^(7)^.

In a study, the calculation of the MCID was performed, which refers to the smallest difference analyzed in an outcome of interest, reported by the patient or measured by the specific instrument capable of identifying changes in the functional status. One of its advantages is to show if relevant changes have occurred in the health status of individual patients. It was identified that changes of 9 points in the global score between assessments should be interpreted by the multidisciplinary team as clinical worsening or improvement of the disease state^(25)^.

As the KPPS scale was developed in another language and in another population (like many other tools), other studies of analysis of evidence of validity, such as analysis of internal structure, should be developed with the aim to increase the instrument validity.

### Study limitations

Despite the limited number of patients included in the study, the test power of 0.90 was considered very high, that is, a high power of the study sample was demonstrated. Furthermore, as pain is a very common symptom with a negative impact on the lives of patients with PD, efforts to deepen the knowledge and characterization of pain in the population of Brazilian patients are important.

### Contributions of the study to the area of nursing, health or public policy

Expanding the degree of evidence of validity of the KPPS instrument for the Brazilian population with PD may allow health professionals to use it in their clinical practice, favoring a more rigorous evaluation to perform multidisciplinary interventions in a systematized and individualized way.

## CONCLUSIONS

The inclusion of 75 older adults and the median KPPS score of 45 points demonstrate the adequate reliability of the instrument, which was evaluated by internal consistency, inter-researcher agreement and stability. It was identified that professionals should consider changes of 9 points in the score between assessments as significant. In the analysis of the convergent construct evidence, the average GPM score was compared with the KPPS score, obtaining a weak but significant correlation coefficient in the three measures.

Based on the findings of this study, the KPPS proved to be suitable for application in clinical practice by the multidisciplinary team, both for the initial assessment and for the follow-up of people with PD. Other validity evidence analysis studies should be developed to further increase the level of evidence for this instrument.
